# A network-based approach to dissect the cilia/centrosome complex interactome

**DOI:** 10.1186/1471-2164-15-658

**Published:** 2014-08-07

**Authors:** Roberto Amato, Manuela Morleo, Laura Giaquinto, Diego di Bernardo, Brunella Franco

**Affiliations:** Telethon Institute of Genetics and Medicine (TIGEM), Naples, Italy; Department of Computer and Systems Engineering, University of Naples “Federico II”, Naples, Italy; Department of Medical Translational Sciences, University of Naples “Federico II”, Naples, Italy

**Keywords:** Cilia, Ciliopathies, Centrosome, Interactome

## Abstract

**Background:**

Cilia are microtubule-based organelles protruding from almost all mammalian cells which, when dysfunctional, result in genetic disorders called “ciliopathies”. High-throughput studies have revealed that cilia are composed of thousands of proteins. However, despite many efforts, much remains to be determined regarding the biological functions of this increasingly important complex organelle.

**Results:**

We have derived an online tool, from a systematic network-based approach to dissect the cilia/centrosome complex interactome (CCCI). The tool integrates all current available data into a model which provides an “interaction” perspective on ciliary function. We generated a network of interactions between human proteins organized into functionally relevant “communities”, which can be defined as groups of genes that are both highly inter-connected and strongly co-expressed. We then combined sequence and co-expression data in order to identify the transcription factors responsible for regulating genes within their respective communities. Our analyses have discovered communities significantly specialized for delegating specific biological functions such as mRNA processing, protein translation, folding and degradation processes that had never been associated with ciliary proteins until now.

**Conclusions:**

CCCI will allow us to clarify the roles of previously unknown ciliary functions, elucidate the molecular mechanisms underlying ciliary-associated phenotypes, and apply our knowledge of the functional roles of relatively uncharacterized molecular entities to disease phenotypes and new clinical applications.

**Electronic supplementary material:**

The online version of this article (doi:10.1186/1471-2164-15-658) contains supplementary material, which is available to authorized users.

## Background

Cilia are specialized evolutionarily conserved organelles protruding from the cell surface of most mammalian cells. The cilium consists of a basal body located under the cell surface, from which the organelle is initially assembled, a transition zone that is important for docking of proteins, and the axoneme. In non-mitotic quiescent cells the mother centriole, a centrosomal component, migrates and docks at the apical cell surface, forming the basal body as a result. The ciliary axoneme, which is nucleated and organized by the basal body, is a cytoskeletal structure, formed by a cylinder of nine doublets of microtubules and in some cases, a central pair of microtubules contained within an extension of the plasma membrane [1].

Cilia can be broadly categorized into two subgroups. Motile cilia, protrude from the surface of cells such as tracheal cells, and promote liquid mobility along the surface. On the other hand, primary cilia, appear as individual non-motile sensory organelles, which, for example, can be found in epithelial cells of kidney tubules and neurons. The ciliary membrane contains ion channels, receptors and other signaling proteins that control axoneme bending for motility and/or sense chemical or mechanical stimuli to transduce internal signals [2]. Primary cilia play a prominent role in development [3] and may even contribute to tissue maintenance and regeneration, as indicated by their presence in stem cells [4].

Mutations in 103 proteins that traffic to the basal body and axoneme of cilia have been causally related to human diseases called “ciliopathies”, disorders which present overlapping phenotypes such as retinal degeneration, skeletal defects, *situs inversus*, obesity, ciliary dyskinesia, mental retardation, CNS malformations and cysts in the kidney, liver and pancreas. Some examples of ciliopathies are Bardet-Biedl syndromes (BBS), Oral-facial-digital type 1 (OFD1) syndrome, polycystic kidney diseases (PKD), Joubert syndrome and related disorders, nephronophthisis and Meckel Grouber syndrome [5].

Model organisms have been extensively used to investigate ciliary functions. High-throughput studies conducted in *C. elegans, C. intestinalis, C. reinhardtii, D. melanogaster, P. tetraurelia, M. musculus* and *H. sapiens* have revealed that cilia are composed of thousands of proteins potentially involved in ciliary function and biogenesis [6–12]. Although much remains to be understood, analysis of the data from the aforementioned studies has begun to resolve the complexity of the cilium. Consequently, the data have also stimulated the creation of many bioinformatics tools, such as those that predict centrosomal genes *in silico* [13], and those which collect, query and analyse these collections of genes in a systematic and organic way. Several databases storing information relevant to centrosome, basal bodies and cilia/flagella are currently available. A non-exhaustive list includes the CentrosomeDB [14] (http://centrosome.cnb.csic.es) the Ciliome Database [15] (http://www.sfu.ca/~leroux/ciliome_home.htm), Ciliaproteome [16] (http://v3.ciliaproteome.org/cgi-bin/index.php), and Cildb [17] (http://cildb.cgm.cnrs-gif.fr/). To date, Cildb is the most comprehensive compilation as it incorporates the largest collection of experiments, including some of those from other databases, in a unified and consistent framework [17]. Nevertheless, although these tools are richly annotated and provide comprehensive lists of genes, they do not display how genes interact with one another and lack information on potential functional networks. Proteins can unquestionably form a variety of functional connections with each other, including a large array of direct and indirect regulatory interactions. These connections can be conceptualized as “networks”, whose organizational structures represent an opportunity to view a given proteome as something more than just a static collection of distinct functions. Indeed, the “network view” is becoming increasingly important in biomedical research and will likely impact applied biology and medical research; protein networks are being utilized to elucidate human diseases on a system-wide level [18] and to predict phenotypes and gene functions [19, 20], to ease drug discovery and development of novel polypharmacology strategies [21, 22].

Previous work has included a preliminary study for a subset of ciliopathy genes [23]. Since some ciliopathies share very similar phenotypes, even with different genetic causes, one could argue that important interconnections occur between genes, and that these interactions play a very important role in pathogenesis. Semi-consistently, the authors found ciliopathy-associated genes to be extremely interconnected. This analysis is however is limited to ciliopathy genes and does not take into account that genes, while not necessarily directly associated with the phenotypes, may play a fundamental and pleiotropic role within the “ciliary interactome”.

In order to resolve this disparity, the list of interactors for each specific gene may now be visualized in CentrosomeDB [14]. Unfortunately this is only possible for one gene at a time, and thus the interactome cannot be analyzed as a whole entity. Furthermore, this database is specifically designed to provide extensive information regarding centrosomal genes and does not display information regarding the relationship between ciliary and centrosomal genes. Indeed, the cilium and the centrosome are two profoundly interplaying organelles, and thus it is possible that some common biological processes are synergistically carried out between them. To overcome these limitations we built a network of curated interactions between human proteins involved with centrioles, centrosomes, basal bodies and cilia to provide a global characterization of the Cilia/Centrosome Complex (CCC) interactome (freely accessible online at http://ccci.tigem.it). By analysing the network we detected functionally relevant “communities”, consisting of groups of genes that are both highly inter-connected and strongly co-expressed. Intriguingly, our analysis unveiled communities significantly associated with specific biological functions that had never been associated with ciliary proteins until now.

## Results and discussion

### Collection of human ciliary and centrosomal genes

We collected 3,502 human ciliary genes from Cildb (http://cildb.cgm.cnrs-gif.fr/), a database dedicated to proteins involved with centrioles, centrosomes, basal bodies, cilia and flagella in eukaryotes which collects the results from 32 high-throughput experiments carried out in 10 different species [17]. Since high-throughput experiments are prone to producing false positive hits, we only kept genes found in the cilium/centrosome in at least 2 different organisms (see Methods). We added 83 genes associated with ciliopathies (as listed in [23]) and, after having removed duplicated entries, we obtained a set of 3,540 genes.

### Definition of the Cilia/Centrosome Complex interactome

We obtained interactions among genes from the “Search Tool for the Retrieval of Interacting Genes” (STRING) database (http://string-db.org), a resource that acts as a “one-stop shop” for all information regarding known and predicted functional links between proteins. STRING quantitatively integrates data from various sources, such as genomic context, high-throughput experiments, co-expression and previous knowledge [24]. We considered two proteins to be “interacting” if: 1) the encoding genes were physically close in the genome and transcribed together or co-expressed, 2) they have been previously shown to interact, and 3) they interact based on the results of high-throughput experiments. For each interaction, STRING collects the evidence available from all sources and assigns a score between 0 and 1, according to the strength and the amount of evidence. A score greater than 0.7 indicates a “high confidence” interaction. In order to obtain a more robust set of interactions, we excluded data from text mining methods (e.g. co-citations) and re-computed the score by only using the other information (genomic context, high-throughput, co-expression and previous knowledge). We obtained 132,873 human high-confidence interactions. After excluding 1,845 singletons (genes with no interactions), we were left with the final CCCI which consisted of 11,608 interactions among 1,695 genes. Notably, 1,593 CCCI genes belonged to a single connected component. The remaining 102 genes were distributed among 27 connected components of two elements and 10 connected components of 3 to 8 elements (Additional file 1: Table S1).

We found CCCI’s structure to be scale-free [25] (alpha = -1.418), which is typical of complex networks in which few genes have a high number of interactions (hubs) while the vast majority only have few.

Among ciliopathy genes, *CEP290*, whose mutations cause a wide variety of distinct phenotypes (namely, Senior-Loken syndrome, nephronophthisis, Joubert syndrome, Bardet-Biedl syndrome, and Meckel-Grüber syndrome) [26], shows the highest number of connections (http://ccci.tigem.it).

We then compared the complete gene set (3,540 genes) with the SYSCILIA Gold Standard (SCGSv1), a list of 303 known confirmed ciliary components curated by the multinational SYSCILIA consortium (http://www.syscilia.org). SYSCILIA Gold Standard contains genes considered to be ciliary only if evidence was published for ciliary localization (including basal body), function in ciliogenesis (including cilium-specific transcription) and involvement in ciliopathies [27]. We found a significant overlap between the two sets, with 70% of SCGSv1 genes (211 out of 303; p-value < 0.01, Fisher’s exact test) present in our collection. Out of the 211 SCGSv1 genes, 76 are present in the CCCI. In our study, we decided to pursue a linear approach, in the sense that the comparison with the SCGSv1 was only aimed at assessing the overall quality of our selection. Since our collection is mainly derived from high-throughput experiments, we cannot rule out the possibility that it may contain both false positive and false negative hits. However, the significant overlap with an independent, well-established and experimentally validated set of genes suggests that our selection criteria are reasonable.

### Genes and communities enrichment analysis

**Figure 1:**
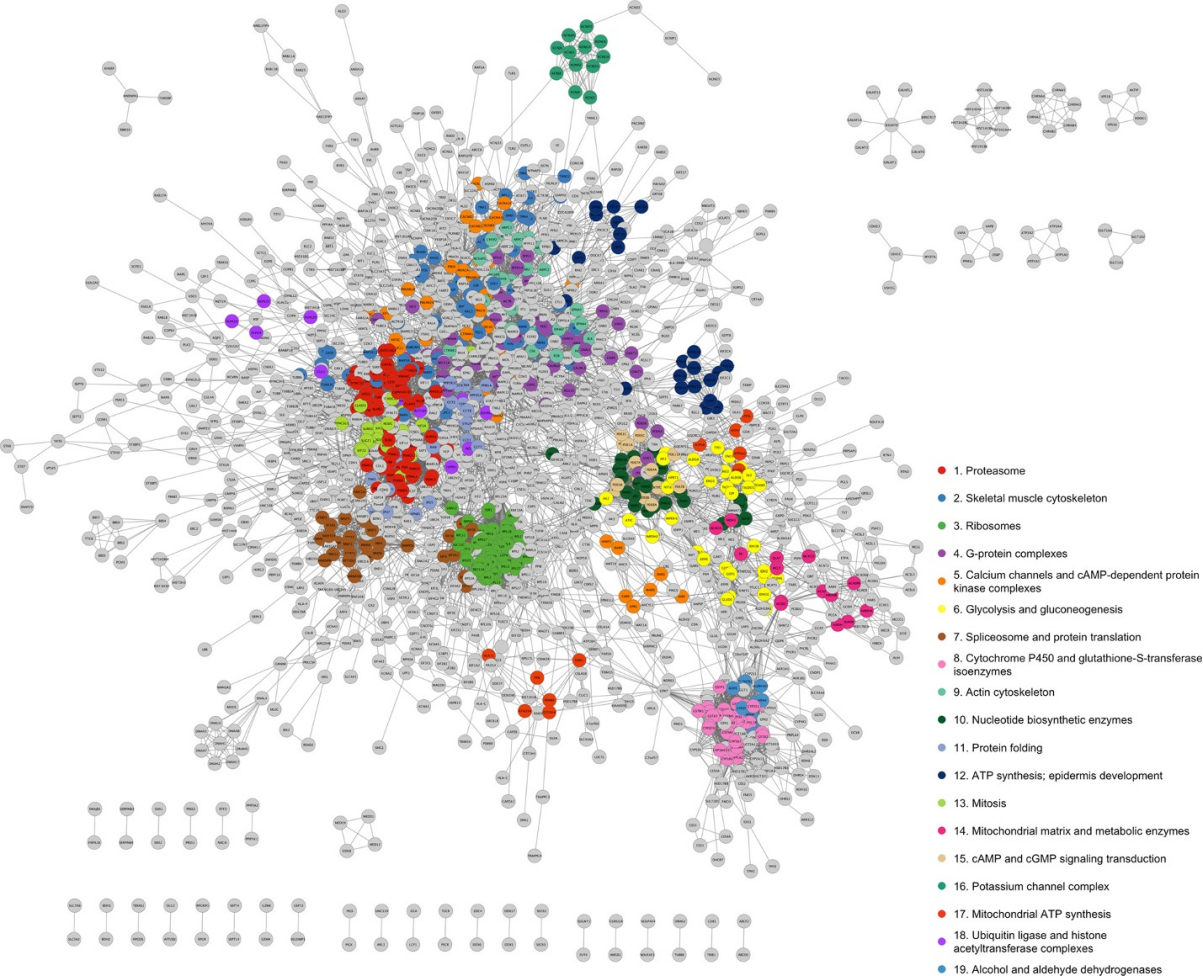
**Gene-wise CCCI.** Genes are represented by circles (nodes), and interactions between them are indicated by lines (edges). Genes included in communities with more than 10 genes are colored, with each color representing a different community as displayed in the legend. On the bottom and on the right the groups of interacting genes that do not belong (i.e. are not connected) to the large connected component are reported in light grey.

The centrosome, basal body and cilium are able to regulate their complex behaviors thanks to a set of proteins that perform different but coordinated functions. We questioned whether or not functional modules could be found within the CCCI, as they may reveal how the network is organized. We searched the network for modules, which are defined as “communities”, i.e. group of genes densely interconnected with each other and connected to few genes outside the group, via a clustering algorithm [28]. We identified 90 communities (Figure 1; Additional file 1: Table S2; http://ccci.tigem.it) containing, on average, 9 genes each (range = 3-65; median = 4). Thirty-nine communities contained at least 5 genes. Figure 1 shows the gene-wise network in which each node is a gene, and each color is referred to as a specific community.

We found with Gene Ontology (GO) analysis that 45 (50%) of these communities were equipped to manage specific biological functions (Additional file 1: Table S2, http://ccci.tigem.it). This percentage increases to up to 74% when only communities composed by at least 5 genes are considered. Twelve communities are composed of at least 20 genes, and each of them is enriched for a specific biological process (Table 1).

**Table 1:** **Communities of the CCCI**

ID	N	Name	Biological process
**1**	**65**	Proteasome	Proteasomal ubiquitin-dependent protein catabolic process
**2**	**62**	Skeletal muscle cytoskeleton	Actin filament capping; cardiac muscle contraction
**3**	**59**	Ribosomes	Translational elongation
**4**	**48**	G-proteins	Signaling pathway transduction
**5**	**35**	Calcium channels and cAMP-dependent protein kinase complexes	Signaling pathway transduction
**6**	**34**	Glycolysis and gluconeogenesis	Glycolysis and gluconeogenesis
**7**	**31**	Spliceosome and protein translation	mRNA processing and translation initiation activity
**8**	**28**	Cytochrome P 450 and glutathione-S-transferase isoenzymes	Metabolic process
**9**	**26**	Actin cytoskeleton	Cell migration and motility
**10**	**24**	Nucleotide biosynthetic enzymes	GTP/UTP/CTP biosynthetic process
**11**	**24**	Chaperones	Protein folding
**12**	**23**	Vacuolar ATPase and keratins	ATP synthesis coupled proton transport; epidermis development

Communities containing at least 20 genes are reported in descending order of the number of components/genes (N). The communities’ ID, name, and the biological process enrichment are reported.

As expected, some communities are enriched in genes encoding cytoskeletal proteins and in molecules involved in signaling pathway transduction, molecules strictly related to cilium motility and proteins that regulate the mitosis process.

Proteins principally belonging to the actin cytoskeleton and proteins preeminently involved in cell migration and motility make up community 9. Components of community 2 belong to the skeletal muscle cytoskeleton like actin, myosin, troponin, tropomyosin, dystrophin and spectrin. Some communities contain genes that encode molecules involved in signaling pathway transduction; communities 4 and 5 contain members of heterotrimeric G-protein complexes, both catalytic and regulatory components of cAMP-dependent protein kinase complexes and different subunits of voltage-dependent calcium channels.

Communities 20, 13 and 27 also correspond to previously-recognized ciliary components and complexes, such as the axonemal dynein complex, proteins regulating the mitosis process, and the Bardet-Biedl syndrome complex (BBSome), respectively. All of these results are consistent with previous findings and represent internal controls that support the validity of our approach [29].

Interestingly, four communities (1, 3, 7, 11) are enriched for processes including mRNA processing, protein synthesis, protein folding and degradation which control the protein expression levels in eukaryotes. Members of community 7 mainly encode mRNA splicing factors, RNA binding proteins, small nuclear ribonucleoproteins (snRNPs), and RNA helicase and protein translation initiation factors; genes encoding structural constituents of ribosomes, translation elongation and termination factors, are components of the community 3. Proteins that promote correct folding of the other proteins, like chaperonin CCT complex subunits and proteins that are involved in ubiquitin-dependent protein catabolic processes, such as ubiquitin, ubiquitin-conjugating enzymes and different 26S proteasome subunits are the main components of communities 11 and 1, respectively.

Functional links between primary cilia and processes that cooperate to control the protein expression levels had never been presented before. Recently, mutations in DDX59, which encodes a member of the DEAD-box-containing the RNA helicase family of proteins, have been associated with Orofaciodigital syndrome [30], a clinically heterogeneous condition comprising different entities, some of which can be ascribed to ciliary dysfunction [31]. Interestingly, patients carrying DDX59 mutations display impaired ciliary signaling [30]. Moreover, a putative RNA-binding protein called SZY-20 has been claimed to traffic to centrosomes and plays a critical role in limiting centrosome size in *C. elegans* [32]. Interestingly, the RNA-binding protein RBM8A, known to be required for mRNA metabolic processes such as splicing, mRNA export and nonsense-mediated mRNA decay, is localized to the centrosome and nuclei [33]. These data suggest that centrosomal and ciliary functions may be associated with mRNA transport and splicing, thus supporting our observations. These results also suggest that the centrosomes and the cilia should be tested as new centers for protein synthesis. This possibility is not far-fetched: it has been suggested that translation initiation factors are localized at centrosomes [34–36], and previous data has proposed that the mRNA binding protein, HuR, stores mRNA in the centrosome and that it controls *de novo* protein synthesis in near proximity to centrosomes via phosphorylation [36]. Moreover, consistent with these observations, studies have reported a correlation between local *de novo* protein synthesis and centrosome formation, supporting the centrosome’s dependence on components of translational machinery [35].

Proteins that are involved in ubiquitin-dependent protein catabolic processes, such as ubiquitin, ubiquitin-conjugating enzymes and different 26S proteasome subunits are the main components of community 1. This non-lysosomal degradation pathway plays a crucial role in biological processes. It not only degrades misfolded and damaged proteins, but also regulates cell-signaling pathways involved in proliferation, adaptation to stress, and regulation of cell size and cell death. Indeed, it has been already shown that, although the ubiquitin proteasome system localizes and can act throughout the cytosol, the catalytic subunit 20S is also localized at the mammalian centrosome [37] Moreover, BBS4 and BBS11, which are involved in Bardet-Biedl syndrome, have been linked to the ubiquitin proteasomal protein degradation pathway [38, 39].

**Figure 2:**
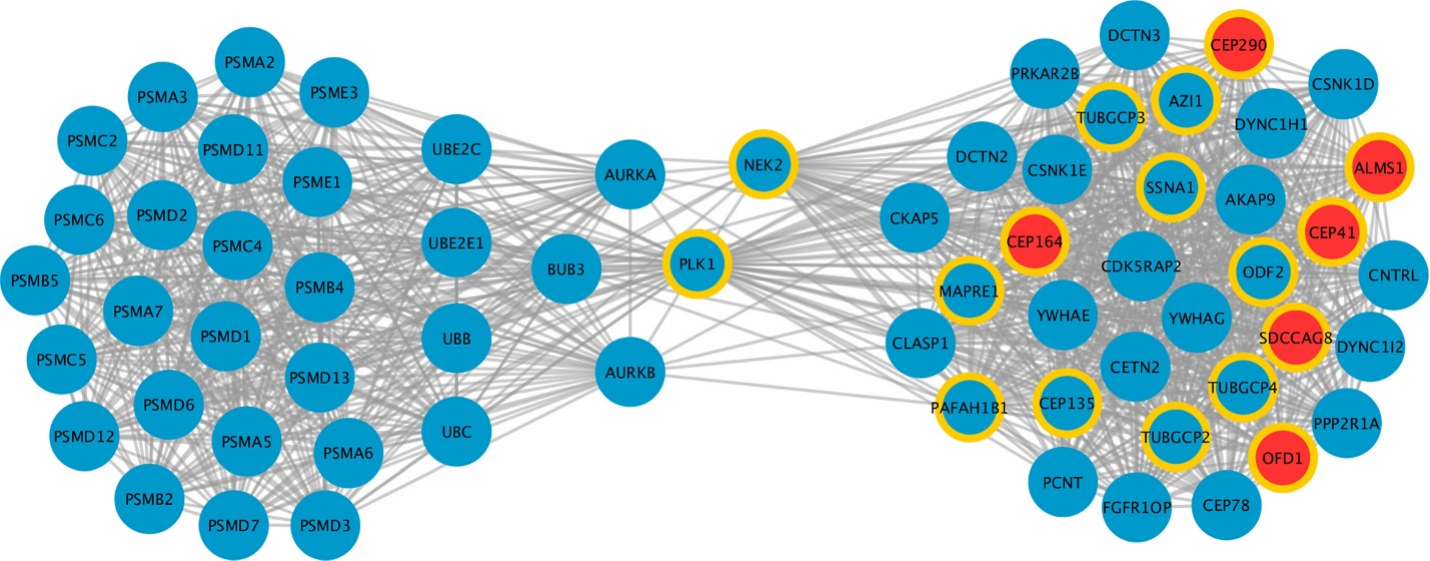
**Detailed representation of the proteasomal community, the largest community identified (community 1).** Each node represents a gene of the community, and grey lines indicate interactions between them. Reported genes associated with ciliopathies are displayed in red (CEP290, CEP164, ALMS1, CEP41, SDCCAG8, OFD1). The yellow border identifies genes also found in the SYSCILIA Gold Standard dataset (SCGSv1).

The “proteasome community” is enriched in ciliary components belonging to the SYSCILIA Gold Standard (SCGSv1) (17 out 76; p-value < 0.01, Fisher’s exact test; Additional file 1: Table S1), and in genes responsible for ciliopathies (6 out 27; p-value < 0.01, Fisher’s exact test; Additional file 1: Table S1) (Figure 2).

We recently demonstrated that *BBS1*, *BBS4* and *OFD1*, a ciliopathy gene belonging to the proteasome community, that codes for a centrosomal/basal body protein and aids in cilia formation [40, 41], depend on proteasomes and mediate protein degradation [42].

Our study now suggests that other ciliary genes, besides OFD1, have a functional connection with the proteasome complex.

CCCI analysis suggests that unexpected ciliary functional modules are involved in the control of protein expression and proteasomal degradation. It is tempting to speculate that the centrosome and/or the cilium could represent a “translasome” organelle in which protein super-complexes link protein synthesis and degradation machineries. However, no data are currently available to support this idea, and further experimental evidence is necessary in order to validate this hypothesis.

In the CCCI, other unforeseen biological processes are supported by genes codifying enzymes involved in metabolic pathways such as glycolysis and gluconeogenesis (community 6) and diverse groups of enzymes belonging to the cytochrome P450 and glutathione-S-transferases superfamilies (community 6 and 8).

Gene products involved in nucleoside triphosphate GTP/UTP/CTP biosynthetic processes and enzymes mediating the acidification of intracellular organelles, such as subunits of vacuolar ATPase (V-ATPase), are members of two of community 10 and 12, respectively. As far we know these biological processes have never been associated with cilia physiology before and further experiments are needed in order to confirm the role of cilia in these processes.

### Super-communities analysis

Genes belonging to different communities differ in the frequency in which they interact with each other and the communities with which they interact. Therefore communities vary in the degree to which they are interconnected and associated with one another. In particular, we expect communities with related genes, e.g. involved in related biological process, to be more likely to be interconnected. We explicitly investigated these types of interactions in order to better elucidate community relationships and obtain a general overview of the processes in which the CCCI is involved.

**Figure 3:**
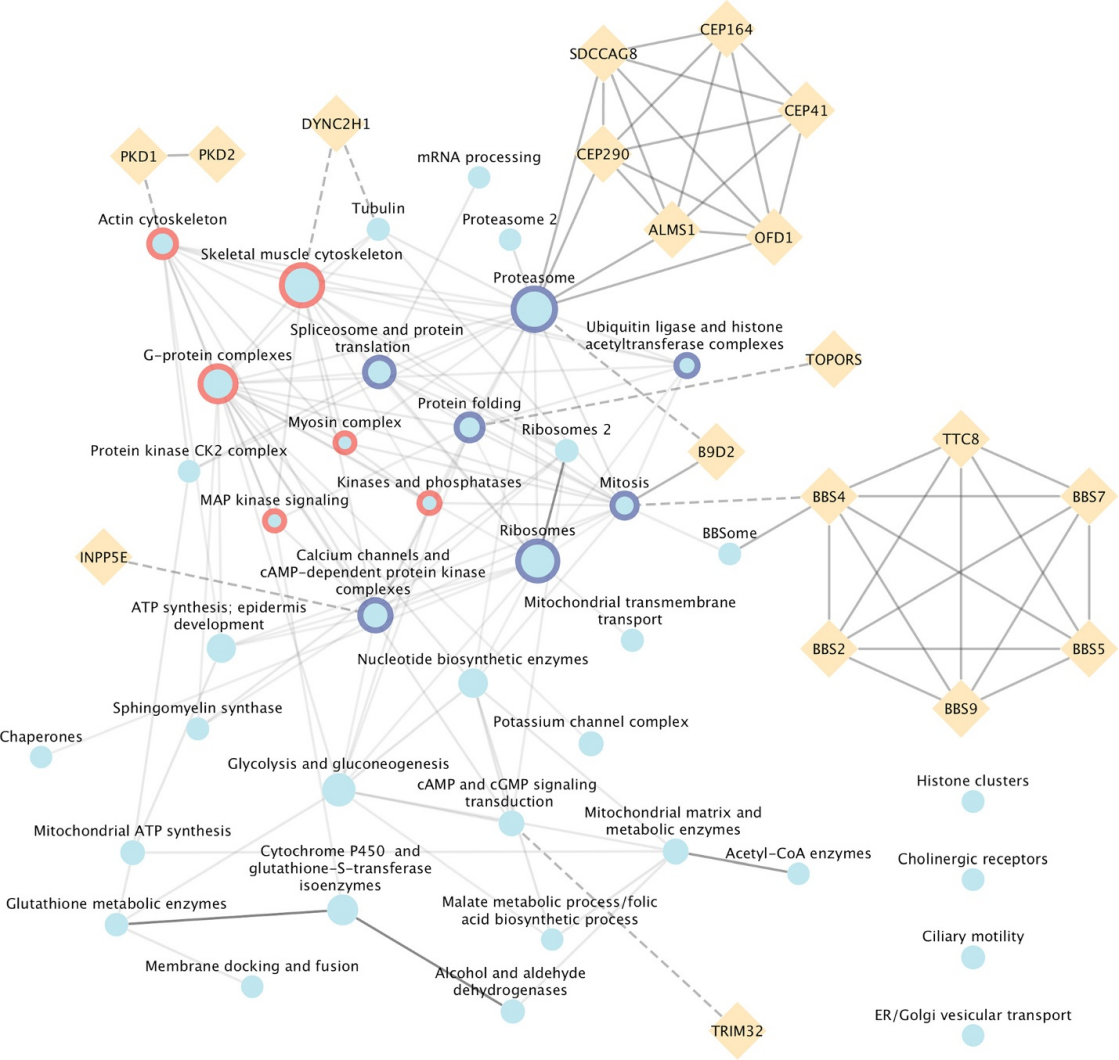
**Community-wise CCCI.** Pale blue circles represent communities. The size is proportional to the number of genes and only communities containing at least 5 genes are reported. Orange diamonds represent genes associated with ciliopathies that belong to or interact with the communities. A solid edge between a gene and a community means that the gene belongs to the community; conversely, a dashed edge means that the gene is connected with at least one member of the community but does not belong to the community itself. The intensity of the edge is proportional to the IS score between the two communities (darker colours correspond to higher scores). Node border colours identify the SC to which the community belongs (dark blue: super-community 1, red: super-community 2).

We calculated the Interaction Strength (IS) between two communities, which is defined as the number of connections between genes belonging to two different communities, divided by the expected number of connections. If the IS is different from 0, then a connection exists between genes belonging to two different communities. Similarly to the gene-wise network, also the community-wise network can also be mapped out by assigning each node to a community. There is an edge connecting two communities only if the IS is different from 0. We obtained a community-wise network composed of 90 communities represented as nodes and 237 interactions. Using the same approach initially used to find communities of genes, we then grouped the communities with at least 5 genes into sets of highly interconnected communities that we call “super-communities” (SCs), i.e. communities of communities. We obtained 2 SCs (Figure 3 and Additional file 1: Table S2). The first SC includes 7 communities with a total of 244 genes and is composed of genes encoding proteins involved in mRNA processing, protein translation, protein folding and ubiquitin mediated degradation (communities 7, 3, 11, 1 and 18). Community 5, which is enriched in genes encoding proteins belonging to voltage-gated calcium channel complexes and cAMP-dependent protein kinase complexes, and community 13, which is enriched in proteins that regulate the mitosis process, also belong to the first SC. On the other hand, the second SC is composed of 6 communities with a total of 155 genes. Three of them are equipped with genes encoding cytoskeletal proteins, or more specifically, components belonging to the skeletal muscle cytoskeleton, proteins belonging manly to the actin cytoskeleton, and myosin proteins (communities 2, 9 and 39, respectively). The three remaining communities are supplied with genes that encode molecules involved in signaling pathway transduction such as heterotrimeric G-protein complexes, phosphorylases and phosphatases and MAP kinases (communities 4, 23, and 30, respectively).

### Proteins sub-localization analysis

In order to distinguish the specific role of the cilium from that of the centrosome, we assigned a potential sub-localization to each gene. We consequently annotated CCCI genes present in “Centrosome:db”, a curated collection of human genes encoding proteins that are localized in the centrosome [43]. Since no similar curated resource exists for cilium, we indicated CCCI genes that were annotated as ciliary in Gene Ontology as “cilium”. This approach led to the annotation of 13% of CCCI transcripts (36 ciliary and 191 centrosomal). In order to achieve better coverage, we annotated the remaining genes taking the specific experiments through which they had been identified into account (see Methods). We further annotated 754 ciliary and 1630 centrosomal genes and left 784 genes labeled as “unknown”. This procedure, based on experimental evidence, allowed us to annotate 75% of CCCI genes overall. The ciliary or centrosomal localization for each protein of the CCCI is reported in Additional file 1: Table S1. One might be tempted to conclude that the different number of genes in the two organelles is indicative of their relative “sizes”. However, these numbers are based on predictions and are not fully supported by experimental evidence, and thus further studies are needed to draw any valid conclusions from this observation.

We thus investigated whether communities were enriched for ciliary or centrosomal genes. Out of 39 communities with at least 5 genes we found 4 communities (10%) to be abundant in ciliary genes (communities 16, 20, 26, 28) and 7 communities (18%) in centrosomal genes (communities 1, 9, 15, 19, 22, 23, 33) (False Discovery Rate (FDR) < 0.05) (Additional file 1: Table S2). Notably, among the ciliary ones, community 20 contained proteins specifically structured for ciliary motility. Conversely, the proteasome community and the actin cytoskeleton community were enriched for the centrosome.

A nuclear localization has been reported in several ciliary genes. From our initial set of 3,540 transcripts we retrieved all genes annotated as “nucleus subcellular localization” in UniProtKB [44]. We found that 402 (24%) of CCCI components also showed nuclear localization. Seven of the identified communities with at least 5 genes (communities 1, 7, 11, 13, 18, 29, 30) were enriched for nuclear genes (FDR < 0.05), although this did not appear to be the case for the CCCI (Additional file 1: Table S1).

Interestingly, we found that some communities contain proteins currently known to be preeminently or exclusively localized to the nucleus. Examples are communities 7, 29, 53, 18 and 32 which contain splicing factors, DNA binding factors, histones and components of a histone acetyltransferase complex, respectively (Additional file 1: Table S1). Community 18, in particular, contains RUVBL1 and RUVBL2, which are components of the NuA4 histone acetyltransferase complex involved in transcriptional activation of selected genes and DNA repair. A functional link between RUVBL1/RUVBL2, DNA repair and ciliopathies has already been established [2, 45, 46].

Other examples of functional links between the centrosome and the nucleus are already present in literature. CHD3 and CHD4, which belong to the nucleosome remodeling deacetylase (NuRD) complex, interact with pericentrin to regulate centrosome integrity [47]. These results, if functionally validated, may reveal new roles for ciliary-associated transcripts.

### Enrichment in transcription factor targets

We finally explored whether communities were enriched in specific transcription factors (TFs) targets. In order to get the most accurate annotation, we combined sequence and expression data. In particular, to keep as many meaningful gene-gene connections as possible, we considered a gene to be a target of a TF if (i) there was a conserved binding site within the gene (plus a flanking region 10Kb upstream and 5Kb downstream) [48, 49] and (ii) it was co-expressed with the TF [20].

**Figure 4:**
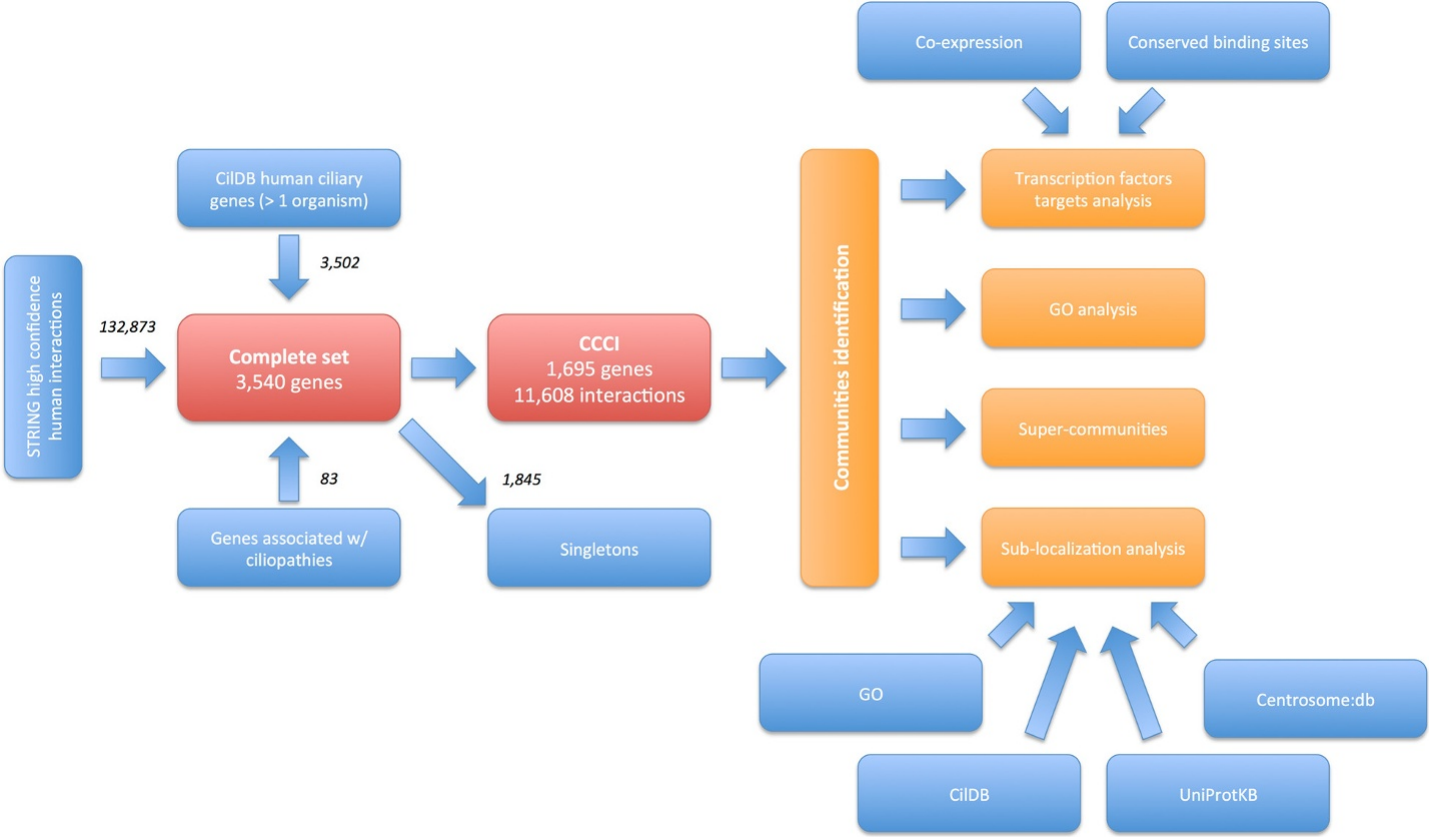
**Schematic representation of the pipeline used for the bioinformatics analysis.** Data sources are indicated in blue, final collections in red and the several enrichment analyses in orange. On the left-hand side steps for the creation of the data sets are reported, while on the right the different enrichment analyses performed are reported.

More specifically, we collected a list of TFs and relative targets with at least one binding site within the genomic region of a ciliary gene from the UCSC Genome Browser. We further refined this list by only retaining targets co-expressed with the TF located in the CCCI. Figure 4 depicts a schematic representation of the pipeline used in this study and also includes the TF analysis. With this analysis we obtained a list of 102 TFs, each with a curated list of potential ciliary targets.

We used this list to explore target density for each community. We found 11 communities to be significantly enriched (FDR < 0.05 and at least 5 targets within the community), in 30 out of the 102 tested different TFs. Additional file 1: Table S2 summarizes the results. Twenty out of 30 TFs are specific to a single community, while 10 are common to more than one community.

As expected, we found that some communities contained members of Regulatory factor X (RFX) transcription factors, which are known to play a conserved role in ciliogenesis throughout evolution [6, 50–52] and the Hepatocyte Nuclear Factor 1 (HNF1), another well-characterized TF involved in PKD [53]. Indeed we identified RFX1 to be specific to community 5, and HNF1A targets in communities 5 and 6. To the best of our knowledge, these are the only transcription factors to have ever been associated with cilia and they can be considered as an internal positive control.

We collected the information from the primary literature and classified the functions and biological roles of the transcription factors that we had identified. Most (54%) of the transcription factors, such as COUP-TF1, AP2REP, GCNF, PAX4, HMX1, HNF1A, LHX3, OCT1, RFX, NFYA, YY1, NKX3, MZF1, GATA1, GATA2 and RUNX1, are involved in developmental processes, and 10 of the 11 communities (communities 2, 4, 5, 7, 8, 9, 11, 12, 15, 20) are enriched in these developmental TFs. Interestingly, 4 of these, MZF1, GATA1, GATA2 and AML1, are regulators of transcriptional events during hematopoietic development.

Communities 2, 4 and 5 host TFs, such as NFAT, AREB6, NF-κB and STAT1, which play a central role in inducible gene transcription during immune response. NF-κB is a TF that is crucial in promoting inflammation through its ability to induce transcription of pro-inflammatory genes [54]. Interestingly we have recently found a functional link between NF-κB signaling and the ciliopathy genes *OFD1*, *BBS1* and *BBS4* both *in vivo* and *in vitro* [42]. Moreover it has been recently shown that mutations in WDR34, a negative regulator of the NF-κB activation pathway, are associated with skeletal ciliopathies and ciliary dysfunction [55].

Communities 4, 5, 11, 15 and 26 include transcription factors involved in cell cycle control such as CREB1, c-MYC-MAX complex, ELK1, E2F1, ER-alpha, FREAC7, CREBP1, RREB1 and MAZR. They represent about a quarter (30%) of the TFs identified.

Communities 4 and 5 also include MYOD, a nuclear protein belonging to the myogenic factors subfamily that regulates muscle cell differentiation and muscle regeneration.

### Web application

Our resource is publicly available as an online tool (http://ccci.tigem.it). The CCCI can be searched by gene of interest and the tool interactively shows information on that gene (e.g. the community to which it belongs to) as well as its interacting genes. It is possible to explore the different communities and their properties.

## Conclusions

Different compilations of centrosome-, basal body-and cilia/flagella- related proteins are available. However, despite the unquestionable value of analyzing genes in the context of their interactions, most of these tools still do not provide a way to realize such analysis.

We integrated information from different sources to provide a comprehensive view on the genes in the cilia/centrosome complex and the interactions among them. By using a network-based approach, we identified communities of genes more closely interacting and showed that most of these communities are enriched in proteins that carry out specific biological processes. Strikingly, we identified several communities related to mRNA processing, protein synthesis, protein folding and degradation. We also showed that some of the communities contain genes that had been, until now, known to be preeminently expressed in the nucleus. Finally, we identified TF targets in some communities, but the roles of these targets and how they contribute to ciliogenesis need to be elucidated. All the results are freely available through an online tool (http://ccci.tigem.it) which allows the user to navigate and explore the interactome.

Our study represents a first attempt to present and analyze the cilia/centrosome interactome, and as such, may have some limitations. For example, as more experimental information becomes available, other gene collections and interactions might be used or included. Furthermore, as typical for many *in silico* approaches, many of the suggested functional links need to be experimentally validated. However, despite its limitations, this work provides new perspectives on the analysis of the cilium.

We believe that the CCCI network that we have generated will represent a valuable tool for the ciliary research community and will help to understand cilia function, to identify potential candidates for gene orphan ciliopathies, and to elucidate unexpected links between cilia and cellular functions.

## Methods

### Data

We collected ciliary genes from Cildb V2.1 [17]. We extracted 8,849 peptides (corresponding to 3,502 unique genes) in the “*H. sapiens* Inparanoid orthologs and filtered best hit” database with “Number of organism with ciliary low confidence evidences” ≥ 2. We then added genes associated with ciliopathies from [23]. High-throughput experiments can lead to false positives and false negatives. By requiring genes to be present in at least 2 different organisms, we expected to drastically reduce the number of false positives. Indeed, this criterion dramatically decreased the number of considered genes considered from 8,879 to 3,502. For symmetrical considerations we did not exclude genes with no evidences of human homologs since lack of statistical association does not necessarily imply that a gene is not present in humans. In fact, 25% of the original set of 8,879 genes are supported by experimental evidence in humans. After selection, this percentage increased to 40%, a proportion very close to what we observe in the SYSCILIA collection (48%) [27]. Albeit not ideal, we reasoned that this procedure overall limits the number of both false positive and false negative in our set of genes.

Gene-gene interactions (GGIs) were extracted from STRING 9.0 [24]. We started from protein-protein interactions present in the *H. sapiens* STRING database and for each of them we recalculated the score as described in [56] but discarded text mining contributions. We then extracted 285,096 protein-protein interactions with scores of ≥ 0.7. Interactions between proteins were thus mapped as interactions between genes, allowing us to obtain 132,873 GGIs. Finally, we only retained 11,608 GGIs occurring among ciliary genes.

The final interactome consists of 11,608 interactions between 1,695 ciliary genes plus 1,845 singletons (ciliary genes with no interactions).

### Statistical and network analysis

We investigated the main categories of genes present in the CCCI by using GO [57]. GO term statistics were calculated in R via topGO [58] using the “weight01” method, considering the set of 1,695 genes present in the CCCI as the background.

Network analysis (connected components and scale-free topology analysis) and visualization were performed using Cytoscape ver. 2.8.2 [59]. Communities and SCs were extracted using the MCODE algorithm as implemented in the “clusterMaker” Cytoscape’s plugin ver. 1.9 [60] with default parameters.

The IS between communities was calculated using the statistical framework R by using the approach defined in [20].

Throughout the study a p-value of 0.05 was considered to be the statistically significant value after false discovery rate correction. All statistical analyses were performed using R ver. 2.12.1 (http://www.R-project.org).

### Sub-localization

Genes’ and, consequently, communities’ sub-localization was obtained by combining different sources. 260 genes out of 383 present in Centrosome:dB [43] were also present in the initial set of 3,540 ciliary genes and were thus annotated as “centrosomal”. 191 of these were in the CCCI. 287 genes were annotated in the human GO cellular compartment ontology as “cilium” (GO:0005929). 112 of them were also present in the initial set of ciliary genes and were annotated as “ciliary”. 36 were in the CCCI.

This approach led to the annotation of 227 CCCI genes. In order to achieve a better coverage, the remaining genes were annotated by considering the specific experiments in which they had been found. In particular, of the 32 experiments reported in Cildb V2.1, 10 were specifically designed to investigate cilia/flagella proteome (hereafter “ciliary group”), and 7 were designed to investigate basal body/centriole/MTOC and centrosome proteome (hereafter “centrosomal group”). For each gene we counted: (A) the number of experiments in the “ciliary group” in which the gene was present and (B) the number of experiments in the “centrosomal group” in which the gene was present. If (A) was larger than (B) plus one (A > B + 1), the gene localization was set to “ciliary”. Conversely, if (B) was larger than (A) plus one (B > A + 1), the gene localization was set to “centrosomal”. If the difference between (A) and (B) was either 0 or 1, the gene was annotated as “unknown”.

The list of nuclear genes was obtained from UniProtKB release 2012_07 (available at http://www.uniprot.org) [44]. We extracted all “reviewed” human genes annotated as “nucleus subcellular localization”, and obtained a list of 4,779 genes. 722 and 402 of these genes were found in the initial set of ciliary genes and in the CCCI, respectively.

The presence of ciliary, centrosomal and nuclear genes in the communities was calculated in R, using the “fisher.test” and “p.adjust” functions, whilst considering CCCI genes as the background.

### Transcription factors analysis

For the TF analysis we used both sequence (binding motif) and expression (co-expression) data. We obtained the sequence information from the DAVID Bioinformatics Resources 6.7 “UCSC TFBS” table, which is based on the “tfbsConsSites” track of the UCSC Genome Browser. In this table, a gene is considered to be target of a TF if it has a binding site for the TF conserved in the human/mouse/rat alignment (i) within 10Kbp-region of 5′ (upstream) end, (ii) within 3Kbp-region of 3′ (down stream) end, or (iii) with in exon/intron region. Any TF without at least one binding site within the genomic region of a ciliary gene (i.e. present in the CCCI) was removed, resulting in a list of 140 TFs.

This list was further filtered by only considering genes/targets that were also significantly co-expressed with the TF according to [20]. To this aim, each TF was converted to the corresponding gene symbol by using the “tfbsConsFactors” track of the UCSC Genome Browser. We removed all targets absent from the CCCI, and eventually all of the TFs ended up with no targets. The final list contained 102 TFs with their relative ciliary targets.

The presence of TF targets in the communities was then assessed using this list, and corrected p-values were calculated in R using the “fisher.test” and “p.adjust” functions, considering CCCI genes as background.

## Electronic supplementary material

Additional file 1: Table S1: Includes information about all the genes included in the analysis; **Table S2** includes information about the communities of CCCI. (XLSX 284 KB)
